# Immunological response after initiation of second line anti-retroviral therapy in HIV patients

**DOI:** 10.1186/1742-4690-9-S1-P11

**Published:** 2012-05-25

**Authors:** Basavaprabhu Achappa, Keerthi Pillai, John T Ramapuram, Satish B Rao, Deepak R Madi, Unnikrishnan

**Affiliations:** 1 Kasturba Medical College, Manipal University, Mangalore, India

## Introduction

Treatment with second line ART is initiated when the first line therapy fails. There is less experience with the immunologic response for second-line ART for adults. Hence this study was done to find out the immunological response after initiation of second line ART by analysis of CD4 counts.

## Methods

This retrospective study was conducted in a tertiary level hospital attached to a medical college that caters to a large number of HIV positive patients. The study population for this analysis included all HIV positive individuals who were undergoing second line ART treatment.The data was collected using semi-structured pre-tested proforma from the hospital records of HIV positive individuals. The immunological response after the initiation of second line ART was analyzed using the CD4 cell counts taken at intervals of 3 months and 6 months after the initiation.

## Results

Out of the 32 patients studied, 27(84.4%) were males and only 5(15.6%) were females. Mean age of the patients was 40.56±6.78 years. The mean CD4 value at initiation was 152.35±142.89cells/μL, which significantly increased to 324.43 ±163.65cells /μLby 3 months after initiation (p value= .000) and to 348.21± 253.57cells /μLby 6 months after initiation.Around 91.3% of patients had a baseline CD4 T cell count <350 cells L-6. After 3 months of therapy, 65.2% of patients and after 6 months 46.2% had a baseline CD4 T cell count <350 cells /μL.The mean weight at initiation was 50.548± 11.37 kg, which very significantly increased to 53.30± 11.1 kg by 3 months of therapy (p = .001, table 1) and to 54.63 ± 10.29kg at the end of 6 months.

## Conclusion

The CD4 counts increase very significantly within the first 3 months of initiation of second line therapy.The rise in CD4 count between 3 months and 6 months is not as statistically significant as the earlier one. Also, there is significant gain in weight within 6 months of initiation of second line therapy.

